# Characteristic Sleep Patterns and Associated Obesity in Adolescents

**DOI:** 10.3390/life12091316

**Published:** 2022-08-26

**Authors:** Ahreum Kwon, Youngha Choi, Sujin Kim, Kyungchul Song, Junghwan Suh, Hyun Wook Chae, Ho-Seong Kim

**Affiliations:** 1 Department of Pediatrics, Severance Children’s Hospital, Institute of Endocrinology, Yonsei University College of Medicine, Seoul 03722, Korea; 2 Department of Pediatrics, Kangwon National University Hospital, Kangwon National University College of Medicine, Kangwon 24289, Korea

**Keywords:** adolescents, sleep patterns, sleep duration, sleep timing, sleep disturbances, obesity, body mass index (BMI)

## Abstract

Poor sleep adversely affects health and may cause obesity. Poor sleep includes short sleep duration, low quality of sleep, and sleep discrepancy. Although most studies have focused on the association between sleep duration and obesity, poor sleep is a significant risk factor for obesity. Adolescents have characteristic sleep patterns which correspond to poor sleep. Adolescents sleep late due to various biological and psychosocial factors; also, they wake up early to be on time for school. This causes them to sleep less. To make up for this sleep debt, adolescents sleep more on non-school days, which causes sleep discrepancies. Therefore, since adolescents have characteristic sleep patterns, an in-depth investigation is needed to identify whether poor sleep is a risk for obesity. This article presents an overview of the characteristic sleep patterns of adolescents, and reviews studies on the association of each sleep pattern with obesity.

## 1. Introduction

Sleep plays an important role in health because poor sleep leads to adverse effects on health. Poor sleep, which includes deprivation, low quality, disruption, and discrepancy of sleep, not only causes sleepiness and decline in performance but is also linked to various health problems [1,2,3,4,5,6,7]. In particular, poor sleep is thought to be associated with obesity and metabolic disease [1], because obesity and sleep insufficiency increase in parallel [2]. Moreover, sleep duration and quality [4,8,9], as well as circadian systems [10] play important roles in metabolic regulation.

Over the last century, most countries have witnessed the prevalence of poor sleep among adolescents [11,12,13,14], which has adversely affected their health [5,6,15,16]. However, adolescents, unlike adults, have characteristic sleep patterns for physiological and psychosocial reasons. The sleep patterns in adolescents are characterized by delayed sleep phases, shorter sleep durations, and larger differences in sleep timing and duration between school and non-school days [17,18], all of which may result in poor sleep. Therefore, in the case of adolescents, the question of whether poor sleep affects obesity should be further discussed in consideration of these characteristic sleep patterns.

This review focuses on the links between poor sleep and obesity in adolescents. The poor sleep patterns in adolescents have been classified into three categories: short sleep duration, delayed sleep phase, and sleep discrepancy between school and non-school days. The effects of these sleep patterns on obesity in adolescents are reviewed in this paper. In addition, the recent findings on adolescent sleep status and sleep recommendations for adolescents are summarized.

## 2. Characteristic Sleep Patterns in Adolescents

Characteristic sleep patterns in adolescents comprise a delayed sleep phase (significant tendency towards later bedtimes and wake-up times), shorter sleep duration, and irregular sleep patterns across the week (discrepancy between weekdays and weekends). These sleep patterns are mainly regulated by biological and psychosocial factors.

### 2.1. Biological Factors

Biological factors affecting sleep patterns include circadian rhythms and homeostatic sleep systems. Circadian timing systems, which regulate the circadian rhythm, have been localized to the suprachiasmatic nuclei of the hypothalamus [19]. Circadian rhythm oscillates over a period of approximately 24 h and controls almost every function of metabolism from the sleep/wake cycle to fasting/feeding behavior [20]. It is a self-regulating and self-sustained clockwork mechanism that synchronizes with the solar cycle. The circadian rhythm is sensitive to light, especially during nighttime [21], and the daily variation of daylight and darkness is the primary synchronizing stimulus for the circadian rhythm [22]. Extended light at the end of daytime or beginning of nighttime moves the circadian rhythm later, and bright light at the end of nighttime or beginning of daytime moves the circadian rhythm earlier [23].

The melatonin hormone, secreted by the pineal gland, is associated with circadian rhythm, and is regulated with changes in the rhythm. Melatonin is rarely secreted during the daytime, but its secretion increases in the evening. Its level is maintained throughout the nighttime and falls when it is time to wake up. Similar to circadian rhythms, the secretion of melatonin is also suppressed by light [24], especially by the short-wavelength of ~460 nm [25], but also by ambient room light [26].

The homeostatic sleep system is dependent on prior sleep/wake conditions, i.e., extended wake attempts to sleep, and prolonged sleep tries to favor waking, regardless of the time of day. The homeostatic sleep system is primarily derived from the slow waves in the sleep electroencephalogram. The slow wave activity is sensitive to prior sleep/wake history, and increases with prolonged waking in a dose-dependent manner [27]. The slow-wave sleep is high at the beginning of the nocturnal sleep episode when sleep pressure is the greatest and declines over the night’s successive non-rapid eye movement episodes [28].

Adolescents show delayed sleep phases, marked by late sleeping and waking up habits [21]. Actigraphic sleep measurements reflect significant delays in sleep onset time with increasing age in adolescents [29]. This is because the circadian timing system changes during puberty due to a positive association of the pubertal stage with later circadian timing [30]. In addition, the release of nocturnal melatonin is delayed in adolescents, leading to a sleep phase delayed by approximately 2 h [31]. These changes reflect a normative developmental course of the circadian clock that shifts toward eveningness around puberty, peaks at around 16 years of age, and shifts back during early adulthood [21,32]. The later circadian timing was found to be related to a longer intrinsic period [33], and a slower accumulation of the “sleep drive” in adolescence [34], making it difficult for most adolescents to fall asleep early. Therefore, the sleep pattern in adolescents is delayed by 2–3 h relative to that in adults [35].

### 2.2. Psychosocial Factors

The circadian timing system undergoes developmental changes during adolescence due to behavioral factors, such as social and scholastic obligations [21]. In adolescence, the increasing demands for academic performance cause adolescents to stay awake longer, resulting in delayed sleep/wake cycles [21]. School-day bedtimes are clearly related to age, as adolescents go to bed later as they grow older [21,35]. Older adolescents have to spend more time on homework or academic activities after school, and as their academic needs increase, they have no choice but to go to bed later. Additionally, the recent development of social media and electronic devices, such as smartphones, encourages nighttime activity and further delays bedtime [36,37]. As the circadian rhythm is sensitive to nighttime light [21], an increased usage of electronic devices delays the circadian rhythm.

### 2.3. Sleep Phases and Duration

These biological and psychosocial factors affect not only the sleep phases but also sleep duration. Most teenagers have to wake up early to reach school on time; hence, they do not get enough sleep. Academic demands/stress and early school start times are the most important contributing factors for sleep deprivation among adolescents [13]. For example, older adolescents are more burdened with social and academic obligations than younger adolescents. Therefore, the older they are, the later the sleep phase is and the lesser is their duration of sleep [29,35,38,39]. However, later school start times are associated with longer sleep duration, primarily due to delayed wake-up times [40,41,42].

Sleep phases of adolescents have been described separately for school days and non-school days as adolescent sleep is typically more variable across a week. The sleep phase is similar among adolescents and adults on school days, but it is drastically delayed by 3 h on non-school days for adolescents, but by only 1 h in adults [43]. This means, physiologically and/or psychosocially, that the sleep phase in adolescents is delayed by 2 h relative to that in adults, and early school start times conflict with this sleep phase delay [21]. This causes a shorter duration of sleep, i.e., sleep debt during school days, and an attempt to catch up over the weekend, leading to greater differences in sleep patterns between school and non-school days [40]. In other words, adolescents tend to sleep longer and later on weekends and holidays to compensate for their physiological sleep pattern changes and sleep debts during the school days. In a meta-analysis, the mean non-school day bedtime was consistently later than the mean school day bedtime, but the significantly later non-school day wake-up time indicated that the mean sleep duration on non-school days was 91.6 min longer than that on school days [35]. The difference between the wake-up times on school days and non-school days was approximately 1.5–3 h for 10–13-year-old adolescents and 3–4 h for high school students [21]. These results indicate that sleep insufficiency on school days results in the creation of sleep debt, and is compensated by more later wake-up times on non-school days. This discrepancy in sleep times between school and non-school days is called “social jetlag”. Social jetlag was observed in all countries, as bedtimes were consistently later and sleep durations were always longer on non-school days than school days [39].

In summary, adolescents prefer late sleep phases, have a shorter sleep duration, and show a large discrepancy in sleep patterns between weekdays and weekends due to biological and psychosocial factors.

## 3. Association between Sleep Duration and Adolescent Obesity

As described above, adolescents sleep for a shorter duration because of the late sleep phase due to the physiological and psychosocial factors as well as the need to wake up early to be on time for school. Several studies investigating the link between sleep duration and obesity in adolescents have found that a short sleep duration increases the risk of obesity [44,45,46,47,48,49,50]. Sleep duration is strongly associated with body weight status [44], as objectively measured sleep duration is inversely associated with body mass index (BMI) [45,46,47,48,49,50]. In a cross-sectional and longitudinal study, the z-score for BMI decreased by −0.22 in the cross-sectional analyses and −0.05 in the longitudinal analysis for each additional hour of sleep [48]. Moreover, sleep duration in childhood affects body weight in adolescence, and age-appropriate sleep patterns in childhood have been found to be associated with adolescent body weight in a longitudinal study [16]. However, not all studies have shown that short sleep duration increases the risk of obesity in adolescents. Some studies have shown a non-significant association between sleep duration and BMI z-score [51,52,53,54,55,56].

Several meta-analyses [1,57,58,59,60,61,62,63,64,65] investigating the association between sleep duration and obesity/overweight status or BMI have shown that sleep duration affects obesity in adolescents (Table 1). Although one study did not specify the method of measuring sleep, three studies analyzed sleep status based on self-reporting and/or by parents, whereas the remaining studies objectively measured sleep duration in addition to self and/or parents’ reporting. Except for one meta-analysis [61], the remaining studies reported that short sleep duration had significantly higher risk of obesity/overweight status and/or increased BMI in pediatric populations [1,57,58,59,60,62,63,64,65]. Short sleep increased the risk of obesity/overweight status by as little as 1.40 times [64] and as much as 2.15 times [59] and decreased the BMI by −0.03 point for every additional hour of sleep [63]. Although one study failed to show a significant effect of sleep duration on changes in BMI, the authors stated that the improvement of sleep duration in children could affect their BMI, nutrition, and physical activity [61]. This is because in some of the studies reviewed by Yoong et al. [61], improvements in sleep duration resulted in weight/BMI loss, and restricted sleep duration increased total calorie intake and sweet/desert intake. Therefore, it is understood that sleep duration can increase the risk of obesity/overweight status in adolescents, despite inconsistency among the results.

Several reasons have been proposed for the increased risk of obesity due to short sleep duration in adolescents. First, short sleep duration causes bad eating habits. Short sleep duration also increases the chance for intake of late-night snacks and food due to long waking times [66,67]. Additionally, short sleep duration is associated with greater responses to external stimuli, such as the sight or smell of food [67]. Children who reduced their sleep by 1.5 h per night for 1 week consumed 134 kcal more per day than those whose daily sleep was increased by the same amount [68]. In adolescents, the total calorie intake was 11% higher due to restricted sleep periods [69]. In addition, several studies have shown that short sleep increases the desire for unhealthy foods. Short sleep duration was associated with an increased intake of fast food and sweets but a decreased intake of fruit and vegetables [70]. The sleep restriction period in adolescents showed a trend of higher consumption of foods with a high glycemic index, such as sweets or desserts, and carbohydrates [69,71]. A systematic review, using data from 33 studies, also showed an association between short sleep duration and less favorable diet quality in children [72]. These psychological eating behaviors due to short sleep duration might be caused by hormonal disturbances related to metabolism and appetite. Second, a short sleep duration leads to endocrine changes causing excess weight gain. In healthy adolescents, the evaluation of the homeostasis model assessment of insulin resistance index increased with an experimental decrease of sleep duration to 4 h per night for 3 consecutive days compared to that with a long-term sleep of 9 h per night [73]. In addition, a short sleep duration is linked to decreased energy expenditure and increased leptin levels [68], leading to further weight gain. Lastly, a short sleep duration leads to physical inactivity due to fatigue and increased screen time [66].

Although it cannot be conclusively established that short sleep duration increases the risk of obesity in adolescents, it is thought to positively influence increased calorie intake, preference for unhealthy foods, altered hormonal levels affecting body weight, and a reduction in physical activity. Therefore, a sufficient sleep duration is recommended to prevent adolescent obesity.

## 4. Association between Bedtime and Adolescent Obesity

Although existing research has traditionally focused on sleep duration as a key contributor to obesity, there is growing interest in evaluating the role of sleep timing, especially late bedtimes [5,74,75,76]. As discussed earlier, the most notable feature of adolescent sleep patterns is the delayed sleep phase. Therefore, it is important to understand whether late bedtimes increase the risk of adolescent obesity.

Bedtime is thought to be a risk factor for obesity [35,74,77,78,79]. The later bedtime/later wake-up time adolescent group was associated with higher BMI than the early bedtime/early wake-up time adolescent group, despite a similar sleep duration [77]. In addition, the later bedtime group positively correlated with the BMI z-score [49,74] with a BMI increase of 2.1 kg/m^2^ for every additional hour of bedtime delay, regardless of sleep duration [78]. Furthermore, the later bedtime/later wake-up time adolescents were 2.16 times more at risk of obesity than the early bedtime/early wake-up time adolescents [15]. Studies have suggested that bedtime could provide a more precise understanding of the association between sleep and obesity [74,80] rather than sleep duration. However, the precise association between bedtime and weight status was unclear in some studies [48,51,54]. The discrepancies in results may be due to differing research methodologies. Although less research has focused on the importance of bedtime compared to sleep duration, bedtime may be an important factor for obesity in adolescents.

Late bedtimes potentially affect dietary behaviors and diet-related health outcomes, which could be the main reason for the increasing risk of adolescent obesity due to late bedtimes. Late bedtimes may also cause increased eating late at night due to staying up late. In addition, although later bedtimes may lead to shorter sleep duration [81], later bedtimes were also associated with less healthy eating behavior, such as less fruit and vegetable intake and more sweetened beverages/fast food intake despite adjusting for sleep duration [5,49,81,82]. The odds of missing breakfast were also significantly higher in cases of late bedtimes [83].

Similar to short sleep duration, late bedtime also affects the amount of physical activity. The late sleep/late wake-up category adolescents had fewer minutes of moderate-to-vigorous physical activity [15,82], while experiencing 48 min more of screen time per day [15] compared to that of adolescents in the early sleep/early wake-up category.

Therefore, further studies are required for a better understanding of the effect of bedtime on adolescent obesity, as this area is a potentially promising target for interventions aimed at reducing adolescent obesity. Large-scale studies are also required to explore the effect of wake-up time on adolescent obesity, as early school start times may be a major cause of insufficient sleep among adolescents.

## 5. Association between Sleep Discrepancy (Social Jetlag) and Adolescent Obesity

As mentioned earlier, most adolescents suffer from “social jetlag.” Adolescents who sleep late biologically and psychosocially are forced to wake up early because of school start times, resulting in sleep deprivation. The sleep debt during school days is compensated by trying to get enough sleep during non-school days. Due to physiologically delayed sleep phases, adolescents sleep later, sleep longer, and wake up later on non-school days. A recent study reported that adolescents obtained an average of 1.4 h more sleep on non-school days than on school days, in addition to shifting bedtimes by nearly 70 min later and wake-up times by nearly 160 min later [84]. Although oversleeping on non-school days may make up for the sleep deprivation during school days, the resulting circadian disruption across the week may lead to obesity in adolescents [85].

It is necessary to examine the effect of sleep discrepancy on obesity in adolescents from two distinct angles. First, whether the sleep duration discrepancy between school and non-school days affect the body weight status of adolescents. Oversleeping or catching up sleep on non-school days can either protect or increase the risk of obesity/overweight status. Research on this is insufficient, and most reports have shown null findings on the harmfulness of oversleeping during non-school days on adolescent obesity [84,86,87]. However, one large-scale cross-sectional study found a significant association between the difference in sleep duration on school days versus non-school days and obesity/overweight status in adolescent females, but not in adolescent males [88]. Second, whether the difference in sleep timing, including bedtime and wake-up time, between school and non-school days increases the risk of adolescent obesity. This difference, called social jetlag, can increase the risk of obesity due to frequent disruption of the circadian rhythm. One study found that overweight/obese adolescents had significantly later bedtimes (average 25 min) during non-school days compared to that of subjects with normal weight, and delayed bedtimes were an independent predictor of higher BMI z-scores [86]. In a large-scale cross-sectional study, social jetlag was associated with increased waist circumference and fat mass index in early adolescent girls, while no such associations were observed in adolescent boys [89]. Another cross-sectional study also showed that social jetlag was positively associated with the BMI z-score and waist-to-height ratios in older adolescents [90]. These findings may be a result of disrupted circadian rhythms caused by differences in sleep timing between school and non-school days. However, it is still controversial, as some studies have also shown that the sleep timing differences between school and non-school days were not associated with body weight status [84,88]. In addition, the results of the link between social jetlag and obesity varied by gender [88,90]. The reason for the difference in results according to gender is unclear, but sociocultural factors and biological factors are thought to have an effect [88,90].

Therefore, it is too early to conclude whether “social jetlag” adversely affects obesity/overweight status in adolescents. However, it is necessary to pay attention to social jetlag in adolescents, and further in-depth research is necessary to understand the effect of social jetlag on adolescent obesity. Social efforts such as adjustment of school schedules including the school start time might be necessary.

## 6. Current Sleep Pattern Status in Adolescents

**Figure 1:**
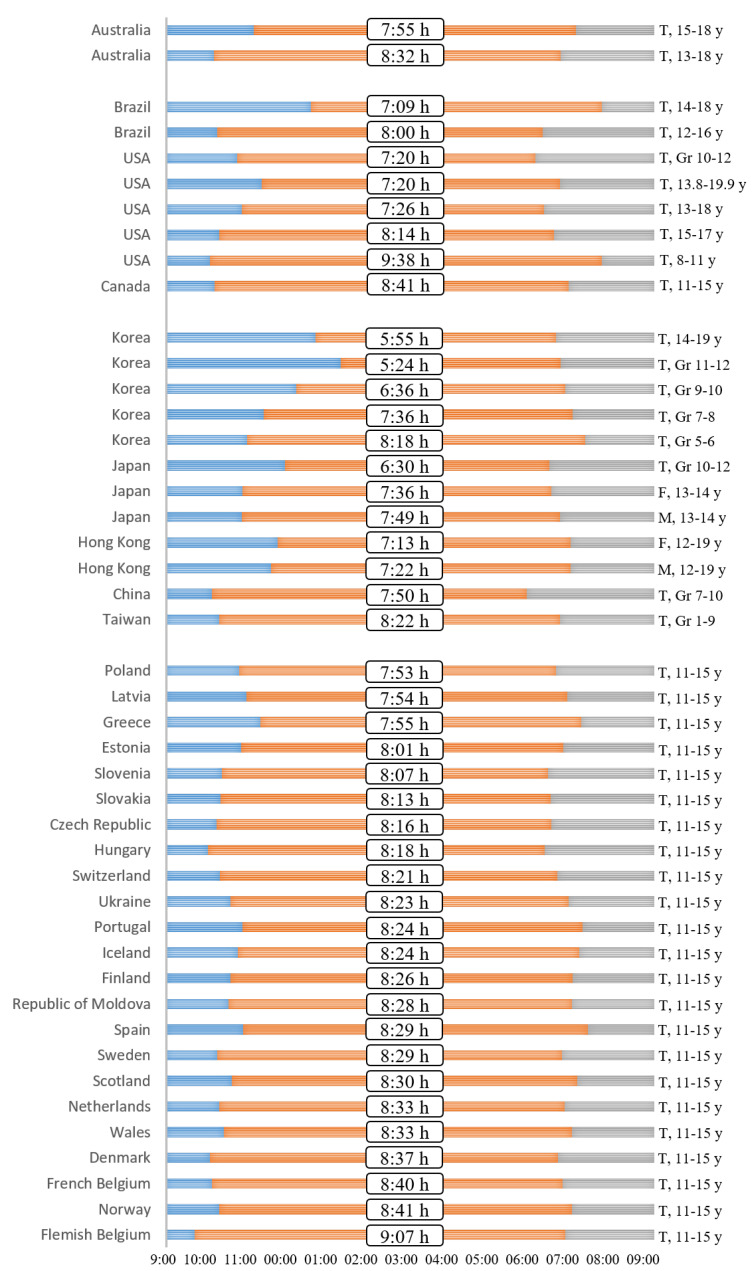
Current global sleep status in adolescents. The European and Canadian data was acquired from Gariepy et al. [39], with permission from Elsevier. Rest of the data was collected from the cited references [13,14,99,100,101,102,103,104,105,106,107,108,109,110,111,112].

Although sleep patterns, such as sleep duration, chronotype, and discrepancy between school and non-school days, are considered to have an important effect on the risk of obesity/overweight status in adolescents, the sleep patterns of adolescents are getting worse. Over the past century, children’s sleep duration has decreased by 0.75 min each year [91], and the rate of change is highest in older children on school days [91,92]. Even though the National Sleep Foundation recommends that adolescents must receive 8–10 h of night sleep [93], the average estimated sleep duration of adolescents aged 10–19 years was approximately 5.5–9.5 h, which varied largely depending on the region (Figure 1). Usually, Asian adolescents sleep less than those in other regions. Asian children sleep 1–2 h less each day than European children and 40–60 min less each day than American children [38]. The difference in sleep duration seems to be influenced by cultural rather than environmental factors (e.g., daylight duration) [35]. Both genetic and socio-cultural differences can also affect sleep duration in adolescents [94].

It is thought that Asian adolescents sleep less because they go to bed later than those in other regions. In a meta-analysis study, the mean Asian bedtime was 11:23 pm, while the North American bedtime was 10:06 pm [35]. The difference in bedtime may suggest the influence of cultural factors, as there is little difference in the geographical latitude [35]. For example, adolescents in China finish school at 8:30 pm [14], which is likely to be later than the end time for schools in North America. Conversely, the wake-up time is relatively consistent across regions and age groups on both school days and non-school days [35]. These results partially explain why Asian adolescents sleep less than those in other regions.

However, only 27% of adolescents in 20 cities in the United States [16] meet the sleep requirements recommendation by the American Academy of Sleep Medicine [95]. According to the reports of the Centers for Disease Control, two-thirds of all adolescents do not meet the recommended sleep requirements, as they sleep for ≤7 h on school days [96].

Several sleep recommendations for children and adolescents have been established (Table 2). Each sleep duration recommendation differs slightly, and it is unclear which sleep recommendations are the most appropriate. In addition, the appropriateness of these sleep recommendations is still uncertain, as ideally the sleep recommendations should be based on ideal sleep duration. The ideal sleep duration to maintain normal mental and physical function is still unknown, because the optimal sleep duration may differ both inter-individually and intra-individually [94]. Different optimal sleep durations, depending on individual cognitive tasks [97] and genetic variations [98], may be required. Therefore, establishing a global standard of optimal sleep is difficult. Furthermore, to the best of our knowledge there are no recommendations regarding the optimal sleep timing or sleep discrepancy across the week. Therefore, from the perspective of preventing obesity in adolescents, appropriate recommendations are needed for proper sleep duration, sleep time and wake-up time, and management of school days and non-school days. This requires better-planned research focusing on sleep patterns in adolescents.

## 7. Conclusions

Adolescents have a characteristic sleep pattern. Appropriate sleep for adolescents is essential, as it plays a crucial role in preventing obesity/overweight status. However, not only is there a lack of appropriate sleep recommendation standards, but also the majority of adolescents do not sleep as currently recommended. Therefore, to prevent obesity among adolescents, it is necessary to encourage them to follow the sleep recommendation protocols as well as to develop a social atmosphere conducive to appropriate sleep and better health. Finally, appropriate sleep recommendation standards for adolescents should be established through further research.

## Figures and Tables

**Table 1:** Meta-analysis studies on the association between sleep duration and obesity in adolescents.

First Author	Number of Articles Reviewed	Study Description	Participant Age (Range)	Sleep Measurement	Outcome Variable	Results
X. Chen [57]	17	Prospective (three cohort, 12 cross-sectional, and two case-control studies)	0–18 years	Self-reported questionnaire, time-diary (another), and wrist actigraphy	Overweight/obesity	Children having recommended sleep duration, those with much shorter sleep duration had a significantly higher risk of overweight/obesity (OR = 1.60; 95% CI: 1.22–2.10). The shortest sleep duration had much higher risk of overweight/obesity (OR = 1.92; 95% CI: 1.15–3.20).
F.P. Cappuccio [1]	12	Prospective	2–20 years	Sleep questionnaires (all)	Obesity	7 of 11 studies reported a significant association between short duration and obesity (OR = 1.89; 95% CI: 1.46–2.43).
H. Ruan [58]	25	Prospective	0–16 years	Parent reported, self-reported, the CDS questionnaire, questionnaire data (TSD-, and accelerometry	Overweight/obesity	By combining data from seven studies (10 independent cohorts), participants in the shortest sleep duration group had significantly higher risk of overweight/obesity as compared with those from the longest sleep duration group (OR = 1.76; 95% CI: 1.39–2.23).
Y. Fatima [59]	22	Prospective	0.5–18.0 years	Parent reported, self-reported, wrist actigraphy, and polysomnography	Overweight/obesity	participants sleeping for short duration had twice the risk of being overweight/obese compared with those sleeping for a longer duration (OR = 2.15; 95% CI: 1.64–2.81).
Y. Wu [60]	15	Prospective	0–18 years	Parent reported, self-reported	Overweight/obesity	Short sleep duration was significantly associated with the risk of future obesity (OR = 1.71; 95% CI: 1.34–2.14).
S.L. Yoong [61]	8	Prospective (two cluster RCTs, three cross-over, one factorial design, and two child-level randomized studies)	Newborns to adolescents		BMI (or zBMI) and dietary intake	Three studies found that multicomponent behavioral interventions involving a sleep component did not change the BMI significantly (n = 360, −0.04 kgm^−2^; −0.18–0.11).
L. Li [62]	12	Prospective	0–18 years	Parent reported, self-reported, time diaries (three studies), and polysomnography	Overweight/obesity	Short sleep duration, based on the random effects model, was statistically associated with obesity (RR = 1.45; 95% CI: 1.14–1.85).
M.A. Miller [63]	42	Prospective	0–18 years	Parent reported, child and parent-reported, wrist actigraphy, polysomnography, and accelerometry	Overweight/obesity	Short sleep was associated with a decrease in BMI per hour of increase in sleep [RR = −0.03; CI: −0.04–−0.01), *P* = 0.001].
Y. Guo [64]	5	Prospective	3–20 years	Parent reported, self-reported, and child and parent-reported	Overweight/obesity	Significant direct relationship existed between short sleep duration and the risk of overweight/obesity (RR = 1.47; 95% CI: 1.26–1.71). Associations also existed between short sleep duration and obesity only (RR = 1.40; 95% CI: 1.01–1.95).
X. Deng [65]	33	Prospective	1–18 years	Parent reported, self-reported, polysomnography, wrist actigraphy, and accelerometry	Overweight/obesity	Overall analysis revealed statistically significant associations of short sleep duration (adjusted RR = 1.57; 95% CI: 1.36–1.81) and long sleep duration with obesity (adjusted RR = 0.83; 95% CI: 0.75–0.93).

OR, odds ratio; CI, confidence intervals; CDS, Child Development Supplement; TSD-Q, Total sleep duration including daytime napping from questionnaire data; RCT, randomized controlled trial; BMI, body mass index; zBMI, standardized body-mass index; RR, relative risk.

**Table 2:** Recommendations for sleep in children.

Reference	References Cited	Recommendation	
		Age	Sleep Needs
Paruthi et al. [95]	No references cited	Infants (4–12 mths)	12–16 h
		Toddlers (1–2 yrs)	11–14 h
		Children (3–5 yrs)	10–13 h
		Children (6–12 yrs)	9–12 h
		Teenagers (13–18 yrs)	8–10 h
Sleep Medicine and Research Center [113], accessed on 30 April 2016	No references cited	0–6 mths	14–16 h
		6–12 mths	14 h
		1 yrs	13.5 h
		2 yrs	13 h
		3 yrs	12.5 h
		4 yrs	11.5–12 h
		5–6 yrs	11 h
		7–8 yrs	10.5 h
		9–11 yrs	10 h
		12–14 yrs	9.5 h
		15–24 yrs	9 h
		25 yrs and older	7.5–8.5 h
Hirshkowitz et al. [93]	No references cited	Newborns (0–3 mths)	14–17 h
		Infants (4–11 mths)	12–15 h
		Toddlers (1–2 yrs)	11–14 h
		Preschoolers (3–5 yrs)	10–13h
		School-age children (6–13 yrs)	9–11h
		Teenagers (14–17 yrs)	8–10 h
Carter et al. [114]	Iglowstein et al. [115] and Crosby et al. [116] cited	0–2 mths	16–18h
		2–12 mths	12–16 h
		1–3 yrs	10–16 h
		3–5 yrs	11–15 h
		5–14 yrs	9–13 h
		14–18 yrs	7–10 h
Heussler et al. [117]	No references cited	1–4 yrs	14–15 h
		5–13 yrs	10 h
		14–18 yrs	8 h
		15–24 yrs	9 h
		25 yrs and older	7.5–8.5 h
National Heart, Lung and Blood Institute [118], accessed on 17 May 2012	No references cited	Newborns	16–18 h
		Preschoolers	11–12 h
		School-age children	10 h
National Sleep Foundation [119], accessed on 17 May 2012	Carskadon et al. [120] cited	Newborns (0–2 mths)	12–18 h
		Infants (3–11 mths)	14–15 h
		Toddlers (1–3 yrs)	12–14 h
		Preschoolers (3–5 yrs)	11–13 h
		School-age children (5–10 yrs)	10–11 h
		Teens (10–17 yrs)	8.5–9.25 h
Harvard Medical School [121], accessed on 17 May 2012	Ferber et al. [122], Dijk et al. [123], and Ohayon et al. [124] cited	Newborns	16–20 h
		1–4 yrs	11–12 h
		Adolescents	9 h

yrs, years; mths, months; h, hours.

## Data Availability

Not applicable.
